# Humpback whale song occurrence reflects ecosystem variability in feeding and migratory habitat of the northeast Pacific

**DOI:** 10.1371/journal.pone.0222456

**Published:** 2019-09-16

**Authors:** John P. Ryan, Danelle E. Cline, John E. Joseph, Tetyana Margolina, Jarrod A. Santora, Raphael M. Kudela, Francisco P. Chavez, J. Timothy Pennington, Christopher Wahl, Reiko Michisaki, Kelly Benoit-Bird, Karin A. Forney, Alison K. Stimpert, Andrew DeVogelaere, Nancy Black, Mark Fischer

**Affiliations:** 1 Monterey Bay Aquarium Research Institute, Moss Landing, California, United States of America; 2 Department of Oceanography, Naval Postgraduate School, Monterey, California, United States of America; 3 Department of Applied Mathematics, University of California Santa Cruz, Santa Cruz, California, United States of America; 4 Ocean Sciences Department, University of California Santa Cruz, Santa Cruz, California, United States of America; 5 Marine Mammal & Turtle Division, Southwest Fisheries Science Center, National Marine Fisheries Service, NOAA, Moss Landing, California, United States of America; 6 Moss Landing Marine Laboratories, San Jose State University, Moss Landing, California, United States of America; 7 Bioacoustics/Vertebrate Ecology, San Jose State University, Moss Landing Marine Laboratories, Moss Landing, California, United States of America; 8 Monterey Bay National Marine Sanctuary, National Ocean Service, National Oceanic and Atmospheric Administration, Monterey, California, United States of America; 9 Monterey Bay Whale Watch, Monterey, California, United States of America; 10 Aguasonic Acoustics, Santa Clara, California, United States of America; Woods Hole Oceanographic Institution, UNITED STATES

## Abstract

This study examines the occurrence of humpback whale (*Megaptera novaeangliae*) song in the northeast Pacific from three years of continuous recordings off central California (36.713°N, 122.186°W). Song is prevalent in this feeding and migratory habitat, spanning nine months of the year (September–May), peaking in winter (November–January), and reaching a maximum of 86% temporal coverage (during November 2017). From the rise of song in fall through the end of peak occurrence in winter, song length increases significantly from month to month. The seasonal peak in song coincides with the seasonal trough in day length and sighting-based evidence of whales leaving Monterey Bay, consistent with seasonal migration. During the seasonal song peak, diel variation shows maximum occurrence at night (69% of the time), decreasing during dawn and dusk (52%), and further decreasing with increasing solar elevation during the day, reaching a minimum near solar noon (30%). Song occurrence increased 44% and 55% between successive years. Sighting data within the acoustic detection range of the hydrophone indicate that variation in local population density was an unlikely cause of this large interannual variation. Hydrographic data and modeling of acoustic transmission indicate that changes in neither habitat occupancy nor acoustic transmission were probable causes. Conversely, the positive interannual trend in song paralleled major ecosystem variations, including similarly large positive trends in wind-driven upwelling, primary productivity, and krill abundance. Further, the lowest song occurrence during the first year coincided with anomalously warm ocean temperatures and an extremely toxic harmful algal bloom that affected whales and other marine mammals in the region. These major ecosystem variations may have influenced the health and behavior of humpback whales during the study period.

## Introduction

Since first being characterized as song nearly a half century ago [1], the cyclic and hierarchically structured vocalization sequences of male humpback whales have been an important subject in behavioral ecology research [2]. The high degree of complexity and variation in humpback song has motivated investigation of its functional roles and temporal evolution. Studies have found evidence that humpback song may function in both intersexual and intrasexual communication [3–5], and it has been hypothesized that song maintains social contact during migration [6] and enables echoic perception [7]. Beyond potential immediate functions of song, its evolution across large spatial and long temporal scales reveals its role in cultural transmission within and between humpback whale populations [8–10].

Early interpretations that humpback song occurs only in breeding habitats have yielded to a growing body of observations of humpback song occurring in feeding habitats [11–19] and during migration between breeding and feeding habitats [6,20,21]. Use of acoustic recording tags that also characterize dive behavior have further shown that song can occur in close spatiotemporal proximity to feeding behavior [16]. This pervasive and complex vocal behavior is essential to humpback whale ecology, yet its occurrence in relation to ecosystem variations in feeding and migratory habitats remains largely unexplored. This study examines occurrence of humpback whale song in a feeding and migratory habitat of the northeast Pacific.

**Fig 1:**
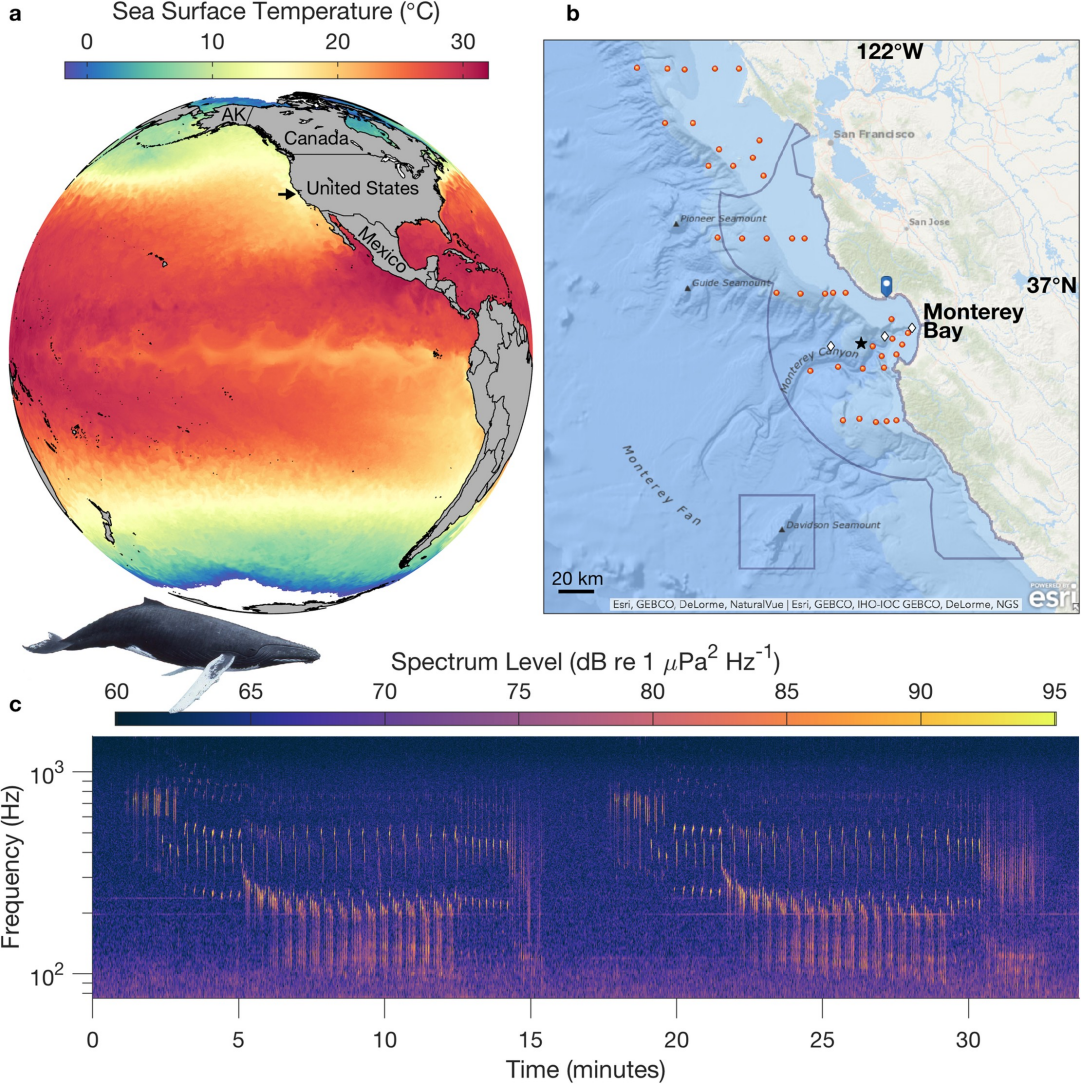
Study region, sampling locations, and focus. (a) The study region is along the northeast Pacific margin, in the California Current System (CCS). Sea Surface Temperature data are from 15-Sep-2016. The arrow indicates the location of Monterey Bay National Marine Sanctuary (MBNMS). (b) MBNMS includes a region of the California coast centered on Monterey Bay, and an offshore region surrounding Davidson Seamount (blue boundaries). The hydrophone is connected to the Monterey Accelerated Research System (MARS) cabled observatory (black star). The MARS node is on Smooth Ridge (36.713°N, 122.186°W, 891 m depth). Sampling locations for primary productivity (white diamonds), domoic acid (blue marker), and forage species abundance (red circles) are shown. (c) Example of two sequential humpback songs recorded through MARS. The spectrogram, with a resolution of 1 second and 2 Hz, was calculated from 12.8 kHz data (decimated from 256 kHz), 6,400 pt. FFT, Hanning window, 50% overlap. Humpback whale image is by Larry Foster.

The study region is the Monterey Bay National Marine Sanctuary (MBNMS), in the central California Current System (CCS; Fig 1A and 1B). This region comprises essential feeding and migratory habitat for two of the fifteen distinct population segments (DPS) of humpback whales identified globally [22]. DPS are named according to primary breeding habitat, however their definition is based on a variety of information including estimates of exchange between breeding areas, geographic patterns of habitat occupancy, distinctions in the ecology of feeding and breeding habitats, and genetic differentiation. Studies examining maternally inherited mitochondrial DNA in these populations indicate natal fidelity to breeding grounds and maternally determined fidelity to feeding grounds [23–25]. Humpback whales that feed off the western United States migrate to breeding grounds off southern Baja, mainland Mexico and Central America (Fig 1A) [26–30]. The basis of this study is a three-year time series (August 2015 through July 2018) of continuous passive acoustic recordings, enabled by a cabled observatory (Fig 1B). The Monterey Accelerated Research System (MARS) cabled observatory sits on the continental slope outside Monterey Bay, California. The cable reaches shore at Moss Landing, the site of a former whaling station [31]. Humpback whale song is prominent in the local soundscape (Fig 1C).

Favorable foraging habitat for baleen whales in the CCS results ultimately from high levels of primary productivity caused by wind-driven upwelling of nutrient-rich water into the euphotic zone [32,33]. The physical signature of coastal upwelling is evident as a cooling gradient between the North Pacific subtropical gyre and the eastern boundary (Fig 1A). Eastern boundary upwelling ecosystems like the CCS are among the most productive of ocean margin habitats [32,34–36]. Beyond elevated primary productivity, favorable foraging habitat for baleen whales results from efficient trophic transfer. This efficiency is partly due to short food chains [32]. Between phytoplankton and humpback whales, the few trophic links are through euphausiids (hereafter ‘krill’) and small schooling fish that consume phytoplankton and microzooplankton grazers. Efficiency is also enhanced through development of foraging hotspots that host dense concentrations of plankton, micronekton, and fish [37–39].

Submarine canyons are particularly important foraging hotspots for krill in the CCS [39]. Monterey Bay coincides with Monterey Canyon, the largest submarine canyon along the western US (Fig 1B). The highest occurrences of krill hotspots in the CCS are associated with canyons between Monterey Bay and Bodega Bay, approximately 200 km further north [39]. Within this region, other forage species for humpback whales exhibit a latitudinal gradient, with anchovy and sardine being more abundant closer to Monterey Bay and maximum abundances of both within the bay [38]. Consistent with this richness in foraging habitat, summer-fall visual sighting data and associated modeling of population distributions off the entire western US show highest densities of humpback whales off central California [40–42].

The overall aim of this study is to understand the occurrence of humpback whale song in a biologically rich feeding habitat that supports relatively dense populations of this whale species. Beyond establishing a foundational description of song occurrence in this ecologically important region, this study aims to examine ecosystem variations that may affect humpback whale foraging ecology, and therefore also song occurrence.

## Methods

### Overview

The study approach begins with detection of humpback whale song from manual analysis of spectrograms, derived from three years of passive acoustic sensing data. Song occurrence is characterized at diel, seasonal, and interannual time scales. At the seasonal and interannual time scales, frequently acquired data from whale watching vessels serve as a proxy for local whale abundance, enabling comparison of song occurrence with statistics of humpback whale sightings in Monterey Bay. The sighting area comprises a portion of the geographic domain for which acoustic modeling defines detectability of humpback whale song at the hydrophone location. At the interannual time scale, ecosystem monitoring data enable characterization of biological factors of importance to humpback whale ecology, which may influence singing behavior. These factors include primary productivity, exposure to a neurotoxic algal compound, and forage species composition and abundance. Hydrographic data and modeling of acoustic transmission loss are applied to consider whether a specific change in geographic patterns of whale habitat occupancy, or changes in acoustic transmission, during an anomalously warm year would have altered song detection at the recording site.

### Acoustic recordings

The acoustic recordings for this study are part of a recently established passive acoustic monitoring project [43]. Recordings were acquired through the Monterey Accelerated Research System (MARS) cabled observatory, in the center of MBNMS (Fig 1B). Since 28 July 2015 MARS has supported nearly continuous recording at a sample rate of 256 kHz using an Ocean Sonics icListen HF–an omnidirectional hydrophone with a bandwidth of 10 Hz to 200 kHz. Data stream directly to the Ocean Sonics *Lucy* software for shore-side recording. In this study we examine the first three full years of recordings, acquired between 1 August 2015 and 31 July 2018. Temporary network outages in the cabled observatory resulted in 94% temporal coverage during the three years.

### Acoustic data analyses

The basis for analysis of humpback song was power spectral density (PSD) computation using the Long-Term Spectral Average (LTSA [44]), adapted for efficient execution in an HTCondor pool of computers [45]. LTSA computations were parameterized for routine processing across two frequency ranges (Table 1). Results from the two frequency ranges were merged to produce spectrograms spanning the frequency range of 0 to 6.4 kHz, retaining high resolution (1 Hz) below 1 kHz, the frequency range encompassing most humpback whale song vocalizations. Custom software written in MATLAB was used to aid manual examination of the entire time series of spectrograms, and to register the time periods of song presence for subsequent analyses, as detailed below.

**Table 1:** Parameterization of LTSA computations.

	Frequency range	
	0–1280 Hz	0 to 6400 Hz
Frequency bin (Hz)	1	10
Sample rate (s^-1^)	2560	12800
Samples per FFT	2560	1280
Time bin (s)	5	5

#### Temporal patterns in song occurrence

The first phase of analysis involved labeling the times of song presence based on manual inspection of spectrograms. Multiple techniques were used to optimize visual detection of song. (1) Spectrogram representations from daily to hourly resolution were simultaneously examined to integrate visual information from longer recording periods covering song sessions with high-resolution detail of song structure. (2) Color scaling for spectrograms at all temporal scales was adaptively constrained to the 1^st^ and 99^th^ percentiles of PSD to maximize visual contrast. (3) The frequency axis of the spectrogram was log-scaled for greater detail in the frequency range where most humpback song energy occurs (Fig 1C). Song was distinguished from non-song vocalizations by requiring evidence of phrase and theme structure [2]; songs were almost exclusively detected within sessions (series of songs, e.g. Fig 1C). Song presence was identified only when it was unambiguous, whether from a single singer or multiple singers. Temporal overlap of multiple singers during many periods of song presence precluded consistent evaluation of pause duration between songs. However, song sessions of single singers consistently showed pauses much shorter in duration than the songs, and song was often nearly continuous within sessions. All time within a song session was labeled as song presence, including pauses. If song signal was weak relative to background for a portion of a song session, such that it was not possible to evaluate consistency relative to the rest of the song session, the uncertain portions were not labeled as song presence.

Analyses of song occurrence at all time scales–diel, monthly, and interannual–normalized song occurrence to the corresponding total recording time. Seasonal variation of song presence was examined for all years separately and as a monthly climatology based on all three years. Because song was absent in summer and peaked in winter, the reference year for the interannual analysis extended from 1 August of one year to 31 July of the next. Diel analysis was based on solar elevation: day > 0°, night < -12°, and dusk/dawn between 0 and -12° [17]. Solar elevation was computed for each minute of recording using the NASA SPICE toolkit for MATLAB [46] with the reference location being the ocean surface above the hydrophone. The proportion of minutes of song presence was computed in 12° solar elevation bins, such that one bin (between 0 and -12°) covered dusk and dawn. Because of the large seasonal variation in the histogram of solar elevation and the need to normalize song time to total recording time within each solar elevation category, this analysis would be biased by including months with very low levels of song occurrence, thereby obscuring diel patterns. Therefore, diel analysis is constrained to the months when most song (76%) occurs: November through January. Data from all three years across these months were aggregated for this analysis. An apparently linear relationship between song occurrence (y) and daytime solar elevation (x) was examined using Model 1 linear regression in MATLAB.

#### Seasonal variation in song length

Using the song presence data extracted in Stage 1, all periods of song were re-examined to quantify the length of individual songs and examine seasonal variation in song length. Consistent with published recommendations [2], the criteria for inclusion in analysis were (1) the ability to distinguish song across its full frequency range, and (2) the ability to define song start and end times consistently from a series of songs in a session, using a marker theme (e.g. Fig 1C). A marker theme for the start of a song was identified from the first song in a session and applied consistently to select start and end times from a subset of clearly defined songs in a session. Song periods containing multiple singers introduced uncertainty in defining individual songs and thus were not included. A total of 1,430 song length measurements were used to quantify variation in a monthly climatology. The entire sample set of song length exhibited a non-normal distribution (Shapiro-Wilk normality test, p < .001). Therefore, to evaluate whether song length changed significantly from month to month, a Wilcoxon rank-sum test was applied. Statistical tests used the Stats package in R, version 3.4.4.

### Acoustic modeling informed by humpback song data

**Fig 2:**
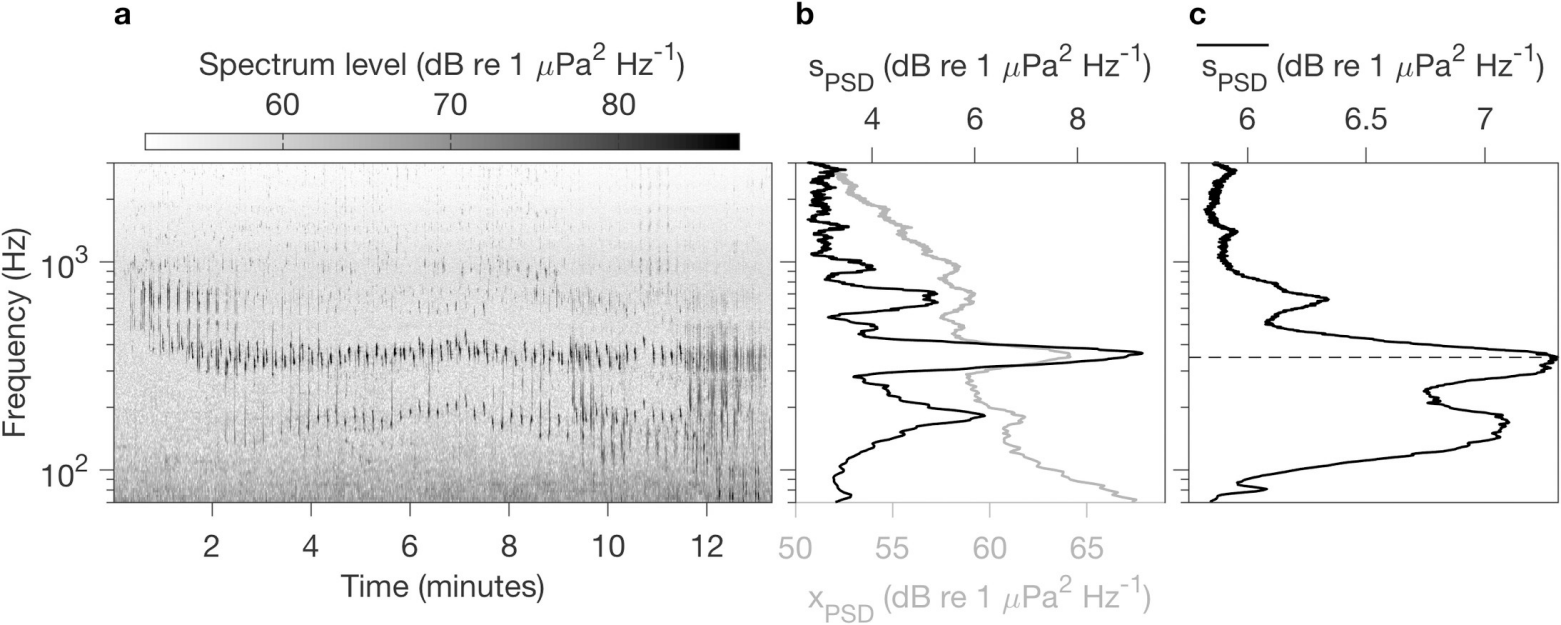
Method of song profile computation. (a) UTC start time for this example song is 16-Jan-2016 11:20:49. The spectrogram, with a resolution of 1 second and 2 Hz, was calculated from 12.8 kHz data (decimated from 256 kHz), 6,400 pt. FFT, Hanning window, 50% overlap. (b) A song profile is defined as the frequency dependent temporal standard deviation of power spectral density (s_PSD_) for a complete song. The temporal mean (x_PSD_) is shown as a gray line for comparison. Mean and standard deviation of PSD were computed over the same time frame, the full duration of an individual song. (c) Mean of s_PSD_ for 429 isolated songs from throughout the 3-year time series. The dashed line marks the maximum at 348 Hz.

Acoustic modeling supports consideration of (1) the source domain for our recordings of humpback song, and (2) the potential influence of variation in habitat occupancy on interannual variation in song detection. To define the frequency at which to model acoustic transmission, a subset of 429 individual humpback songs was selected for analysis. Selection required (1) signal strength sufficient to accurately represent song frequency content, i.e. avoiding faint song signal from distant whales, and (2) absence of extraneous sound sources that would contaminate analysis of song frequency content. For each song a high-resolution (1 second, 2 Hz) LTSA was computed, and for each LTSA of a full song (Fig 2A) the frequency-dependent standard deviation of power spectral density defined a “song profile” (Fig 2B). Comparison of the temporal mean and temporal standard deviation of PSD illustrates why standard deviation (s_PSD_) is a better measure of a song profile (Fig 2A and 2B). It provides greater distinction of the frequencies that song units occupy, it is not dominated by variation in the background, and it is less susceptible to tonal anthropogenic noise. The frequency of the maximum in mean s_PSD_ from all songs defined the frequency at which received levels at the hydrophone would be modeled (Fig 2C).

Acoustic transmission loss at 350 Hz was calculated from a wave-theory parabolic equation model that accounts for absorption in both the water column and the bottom, scattering in the water column and at the surface and bottom, geometric spreading (spherical and cylindrical), refraction, and diffraction [47]. Specification of regional ocean temperature and salinity was based on the September climatology from the US Navy Generalized Digital Environmental Model (GDEM). Bathymetry was specified at 250 m resolution. The source level of 175 dB re 1 μPa was based on published in situ measurements [48] and used to compute received levels at MARS, to characterize the spatial domain around the hydrophone over which song would be detectable under different noise conditions. Sound source depths were specified as 10, 20, or 30 m. Because the results were very similar for these depths, mapped results from the shallowest sound source (10 m) are presented to include results for sound sources in shallow water close to the coast (inner ~1–5 km), which can be important habitat for humpback whales in Monterey Bay.

### Visual sighting data

To interpret temporal variations in humpback song detection in relation to visual sightings, this study used sighting data from commercial whale watch operations (Monterey Bay Whale Watch). The primary advantages of these data are frequent sampling, with visual surveys averaging 326 ± 21 days per year during the period of this study, and consistent coverage of an area near the hydrophone (southern Monterey Bay within ~15 to 35 km from the hydrophone, Fig 1B). These were not systematic surveys, but rather represent number of whales sighted on targeted trips, with destinations often being determined by where whales were known to have been sighted already. However, we expect that lower frequency (seasonal, interannual) patterns in regional presence will be reflected in statistics computed from these frequent observations. Although abundance of adult male humpback whales would be most relevant to song, the sighting data cannot support this degree of specificity. Since the whale-watching record variably included 1–3 vessels making 1–3 partial or full-day trips on each day, daily whale counts were normalized to a constant unit of effort, measured as humpback whales sighted per half-day trip. For comparison with humpback song occurrence at interannual time scales, temporal averaging was constrained to the annual period of song detection, September of one year through May of the next year.

### Ecosystem data

#### Physical data

Variations in sea level reflect changes in the depth of nutrient source waters, while variations in wind-forced upwelling define the primary transport mechanism of nutrients from depth into the euphotic zone. These two physical variables are key predictors of the primary productivity supporting forage species assemblages that baleen whales ultimately rely upon [32,36,37]. Sea level anomaly (SLA) data were from the Integrated Multi-Mission Ocean Altimeter Data for Climate Research sea level data set, spanning 1993 through 2018 [49]. SLA data were subset for the CCS according to analytically defined boundaries [50] (23°N to 43°N within 800 km of the coast) and averaged spatially and seasonally, then the seasonal climatology was removed to emphasize interannual variation. Wind-forced upwelling was examined using the coastal upwelling index [51] for 36°N, 122°W (near Monterey Bay) obtained through the NOAA ERDDAP server [52].

While SLA data do not extend onto the continental shelf, ocean temperature data do and can thus describe physical variations within humpback whale foraging habitat near the coast, as well as pycnocline depth and strength that can affect acoustic transmission from shallow sound sources to a deep receiver. Regional ocean temperatures were examined using remotely-sensed and in situ data. Satellite data were Version 4.1 Multi-scale Ultra-high Resolution (MUR) sea surface temperature (SST) monthly anomaly, 2002 through 2018, produced by NASA Jet Propulsion Laboratory and accessed through the NOAA ERDDAP server [52]. In situ data were from three sources. The first was a glider section time series along California Cooperative Oceanic Fisheries Investigations (CalCOFI) Line 67, which extends offshore of Monterey Bay. This glider time series, 2007 through 2018, is part of a larger glider research program in the CCS [53]. Both satellite and glider time series were processed to seasonal anomalies. Pycnocline variation was examined using continuous observations from a CTD string on mooring M1, at the mouth of Monterey Bay (middle white diamond in Fig 1B) and periodic CTD cast data from station C1 (nearshore white diamond in Fig 1B). These locations are part of a time series observation program [54]. Stations in the bay are most relevant because our focal question is whether preferential habitat occupancy within Monterey Bay during a warm anomaly would affect acoustic transmission to the MARS hydrophone.

#### Biological data

Biological data included primary productivity, concentrations of the neurotoxic algal compound domoic acid (DA), and forage species abundances. Primary productivity measurements were from a time series program [54] regularly sampling three stations within and outside of Monterey Bay (white diamonds in Fig 1B). From the results of carbon uptake incubations, water column primary productivity was computed by integrating across the depth range spanning all light levels of incubation: 100, 50, 30, 15, 5, 1, and 0.1%. The CCS is prone to harmful algal blooms (HABs) that result from food web transfer of the biotoxin domoic acid (DA) [55–57]. DA is produced by several species of the diatom genus *Pseudo‐nitzschia* and can cause illness, heart and brain damage, reproductive failure, and mortality in marine mammals, including baleen whales [55,58–61]. For this study we use weekly measurements of particulate domoic acid (pDA) from Santa Cruz Wharf (location in Fig 1B). Quantification of pDA was by liquid chromatography‐mass spectrometry [62]; data were obtained through the Southern California Coastal Ocean Observing System data server [63].

To examine forage species consumed by humpback whales, standardized abundance data were derived from the NOAA-NMFS Rockfish Recruitment and Ecosystem Assessment Survey (RREAS). The RREAS conducts an annual mid-water trawl survey during late April through mid-June to assess ocean conditions and the abundance and distribution of epipelagic micronekton off California (1983-present). The RREAS samples a variety of epipelagic micronekton utilized by mid and upper trophic level predators, including krill, pelagic juvenile rockfishes (*Sebastes spp*.) and northern anchovy (*Engraulis mordax*) [64]. Micronekton samples were collected at fixed sampling locations (Fig 1B) during night using a modified Cobb midwater trawl with a 9.5-mm cod-end liner; 15-minute tows were made at each station with a headrope depth of 30 m [64]. After each haul, all taxa were enumerated, partitioned into either young-of-the-year (YOY) or adult classes as necessary, and relative species abundance was measured as catch-per-unit-effort (CPUE) per station. For a description of the spatial distribution and temporal variability of numerically dominant taxa, community structure, and an assessment the variability of biodiversity, see [38], [65], and [66], respectively. Due to their importance in the diet of humpback whales [67], we quantified the variability of abundance for total krill and northern anchovy during 2015–2017 using data from all net-tow stations [68,69] (locations in Fig 1B). Anomalies of abundance were calculated from the spatial mean CPUE across all stations sampled between Bodega Bay and Pt. Sur (Fig 1B) and were standardized according to the long-term mean and standard deviation over 1990–2017 [64,68]. Forage species data are available from NOAA [70].

In examining ecosystem variations in relation to interannual changes in song occurrence, leading temporal lags are appropriate because ecosystem conditions preceding the annual (fall) rise of singing behavior could influence whale population dynamics, foraging ecology, and behavior. Beginning with forcing of primary productivity by wind-driven upwelling, we quantify cumulative upwelling between the annual onset in spring through the end of the calendar year. For the three winters examined, strong downwelling caused by winter storms consistently ended by March. Therefore, a consistent measure of cumulative upwelling spanned March through December of each year. This same annual period was used for mean sea level in the CCS, spatially and temporally integrated primary productivity in the Monterey Bay region, and particulate domoic acid (pDA) concentrations. For pDA we quantify the annual temporal span of detection, maximum concentrations, and a potential exposure metric integrated annually by summing the product of each concentration measurement and the time period it represents (1 week). Forage data are from spring/summer of each year and provide an indicator of the overall condition of forage species populations within the central CCS [71]. To describe ecosystem physical patterns from satellite and in situ temperature data, the averaging period was constrained to the second half of the year because glider sampling was missing for the second quarter of one year, and the consistently sampled period of the third and fourth quarters spans late-summer foraging and the annual period when song begins and rises to its annual peak.

## Results and discussion

### Humpback song detection

**Fig 3:**
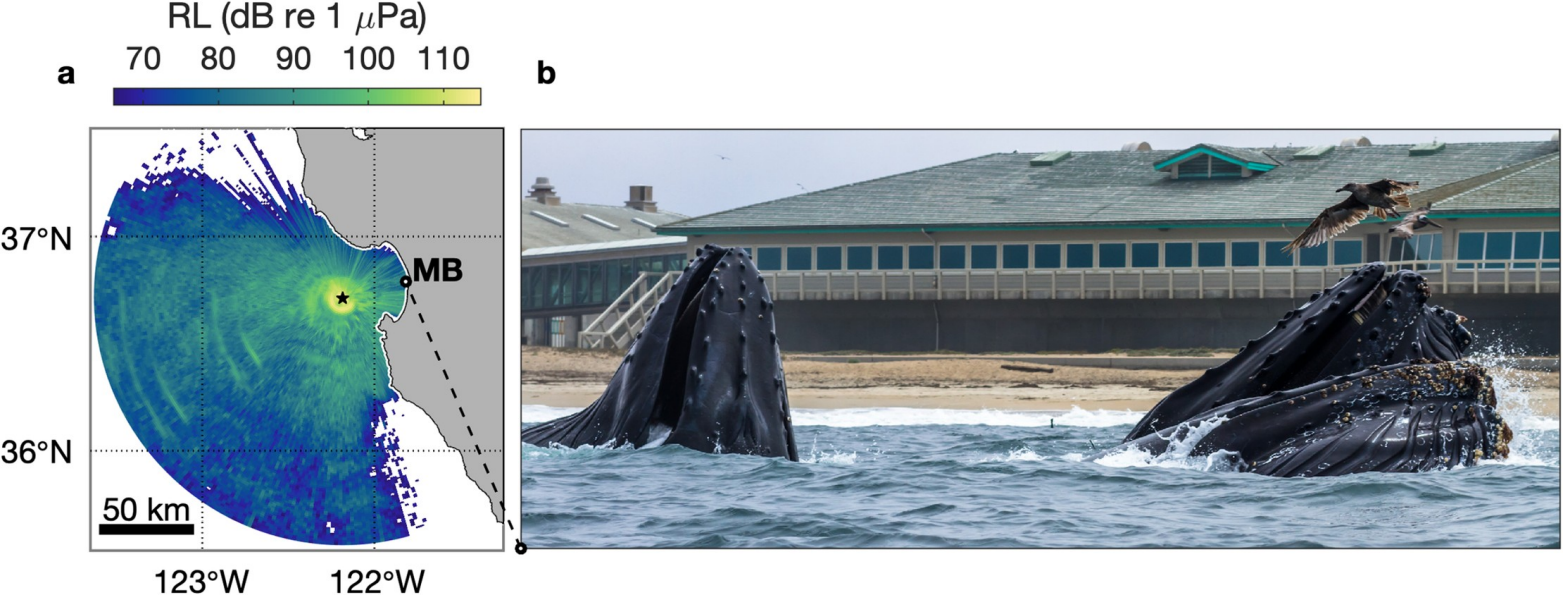
Humpback whale song detection. (a) The map represents received levels (RL) at the MARS observatory for a sound source at 10 m depth having a frequency (350 Hz; Fig 2) and source level (175 dB re 1 μPa, peak-to-peak) representative of humpback song [48]. The MARS hydrophone location is indicated by the black star. (b) Example of humpback whales foraging in nearshore habitat near the head of Monterey Canyon (Fig 1B); photo by Alan Gonzalez is from the location indicated in the map, 25-Jul-2014.

The region around the hydrophone from which humpback song would be detectable under different levels of background noise is characterized from received level for a sound source having a frequency (350 Hz; Fig 2) and source level (175 dB re 1 μPa, peak-to-peak) representative of humpback song [48]. Model results for 10, 20, and 30 m deep sound sources showed very similar patterns. We show the results from 10 m (Fig 3A) because the 10 m isobath reaches closest to the coast, and it is important to evaluate reception of humpback song signal from throughout the bay, including nearshore habitat (Fig 3B). These results show that under relatively loud background noise conditions (110 dB re 1 μPa) humpback song would be detectable at MARS only if the singing whale was within ~ 20 km of the hydrophone (Fig 3A). Under relatively quiet background noise conditions (70 dB re 1 μPa) humpback song would be detectable at MARS if the whale was within a much larger area, including most of Monterey Bay (Fig 3A). Our humpback whale sighting data are from southern Monterey Bay, which is within the domain of MARS acoustic detection of humpback song under background noise conditions below ~ 97 dB re 1 μPa (Fig 3A). Thus, comparison of sighting and song data is appropriate.

### Time series overview

**Fig 4:**
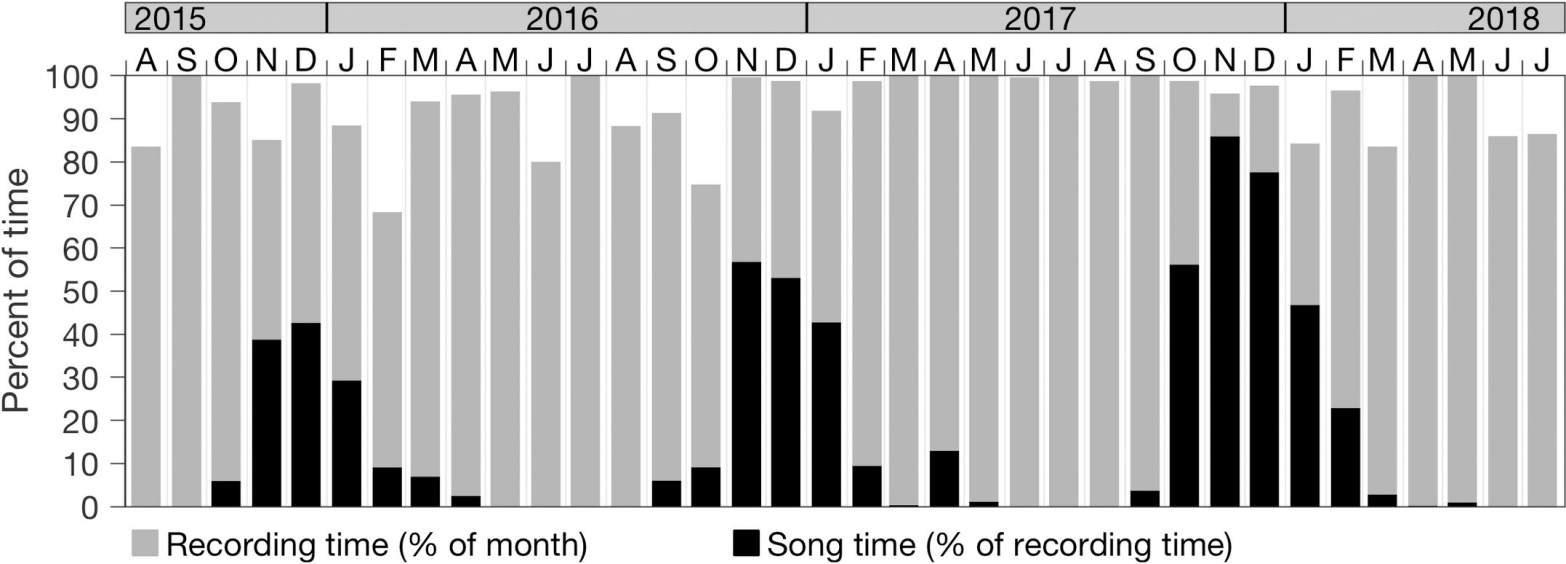
Time series overview. Humpback song presence during the first three years of MARS recordings (black bars). Monthly values represent the percent of recording time during which song sessions were detected, whether from single or multiple singers (see methods). Also shown is the percent of time that was recorded in each month (gray bars).

The three-year record of humpback song detection shows large seasonal and interannual variations (Fig 4, black bars). Song began during fall of each year (September–October) and rose steeply to a peak in November or December. The temporal coverage of song reached a maximum of 86% during November 2017. Song occurrence dropped steeply after January of each year, exhibiting highly variable levels during February through May. Song was not detected during June through August in any year. Integrated over the annual cycle (August through July), total song occurrence increased by 44% between the first and second years, and by 55% between the second and third years. Considering the recording coverage throughout the time series (Fig 4, gray bars), song occurrence patterns described at monthly resolution for the full time series are robust. Since seasonal variation informs analyses of diel and interannual variation, we begin with describing seasonal aspects of song occurrence, then diel and interannual variability. At each temporal scale, we evaluate hypotheses for observed patterns of song occurrence.

### Seasonal

**Fig 5:**
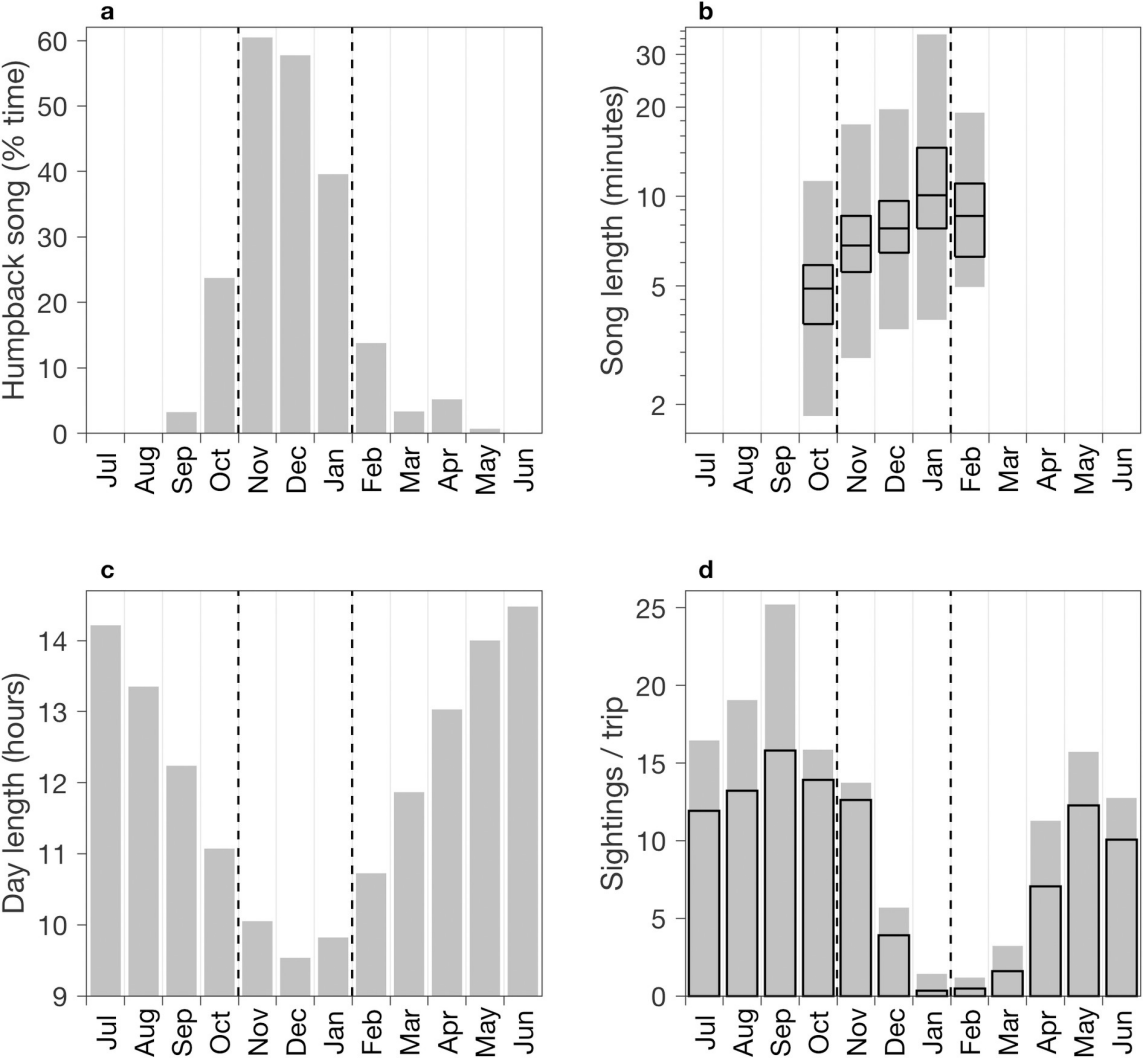
Seasonal variations. Monthly song statistics are based on data from all three years of recording (Fig 4): (a) percent of recording time during which humpback song sessions were detected and (b) song length. Song length is represented as a nonparametric box plot, showing only months having sufficient data (defined as more than 5% of monthly recording time); the range is shown in gray, and black boxes are bounded by the 1st and 3rd quartiles and bisected by the median. (c) Monthly mean day length. (d) Monthly mean number of humpback whale sightings per half-day whale-watching trip in southern Monterey Bay based on sighting data from the three years of this study (solid gray bars) and all 16 years (2003–2018; black outline bars). Sighting data were acquired on 326 ± 21 days per year during the period of this study. Vertical dashed lines in all panels delineate the annual peak in song during November–January.

Monthly mean song levels show that 76% of all song occurs within one quarter of the year, November through January (Fig 5A). Between the start of song in fall through the end of the annual peak in January, song length increases significantly each month (p < 0.01, Wilcoxon rank sum). This increase is evident in minimum, maximum, and quartiles of song length (Fig 5B). Median song length increases from 4.9 minutes in October to 10.1 minutes in January. The annual peak in song coincides with the winter trough in day length (Fig 5C), and with sighting-based evidence of population movement (Fig 5D). Following high levels of humpback sightings during summer and through the start of the song peak in November, sighting levels drop steeply during December and January (Fig 5A and 5D). This seasonal pattern in sightings during our study is consistent with that based on the full sighting record, 2003 through 2018 (Fig 5D). Our sighting data cannot define how much of the humpback population remains in the region, and within the detection range of the hydrophone, after leaving Monterey Bay. However, these indications of population movement during November–January (Fig 5D), coincident with the annual song peak (Fig 5A) and day length trough (Fig 5C), are consistent with seasonal patterns of migratory and vocal behavior driven by changing ecosystem conditions leading into the breeding period.

The South Pacific analogue to the CCS is the Humboldt Current System (HCS). Like the CCS, physical distinction of the HCS caused by coastal upwelling is evident in the cooling gradient between the subtropical gyre and the eastern margin (Fig 1A), and it is important habitat for humpback whales. In the HCS the first continuous acoustic monitoring off southern Chile also demonstrated humpback song occurrence in feeding habitat [17]. Song was detected throughout the 130-day study during February–June 2012. Although this study did not sample a full year, it revealed increasing song occurrence during austral fall and a maximum in winter (June), consistent with our results. Studies of humpback song in the western and eastern North Atlantic also exhibit seasonal patterns consistent with those observed in the northeast Pacific during our study. A decade of acoustic monitoring west and north of the British Isles, eastern North Atlantic, detected humpback song during October–April and equatorward progression during January–March, consistent with migration to low-latitude breeding habitat [13]. Research in Stellwagen Bank National Marine Sanctuary, western North Atlantic, showed increasing song occurrence during the fall–winter transition, when males prepare to migrate to low-latitude breeding grounds [15]. Seasonal variation in hormone levels and associated neurological changes have been hypothesized as a regulatory factor in the seasonal variations in humpback song occurrence [14,15,72]. Measurements of testosterone levels in blubber samples from North Pacific adult male humpback whales show minimum levels in summer, maximum levels in winter, and an increasing trend during fall [73], supporting the hypothesis of hormone levels as a regulatory factor in singing behavior.

Seasonal increase in the length of song sessions has been reported from analysis of recordings from the northwest Atlantic [15]. To our knowledge, seasonal variation in the length of individual songs has not been examined in previous studies. The steady seasonal increase in song length we observed motivates consideration of possible causality. Primary hypotheses include learning, which is the basis for song evolution [8–10], and behavioral influence of seasonally rising hormone levels [73]. Singing behavior during periods of heightened song activity within a population may lead to increasing song length through cognitive development. Conditions that covary with population density, such as prevalence of song activity and diversity of song unit types, may also play a role in learning and associated changes in song attributes (complexity, length). Supporting relatively high population densities, favorable feeding habitats such as Monterey Bay may enhance density-dependent influences on learning. Seasonally increasing hormone levels may affect singing behavior and song attributes, including length. Beyond the scope of this study, how song complexity and composition change with increasing song length is of interest in understanding humpback whale behavior.

### Diel

**Fig 6:**
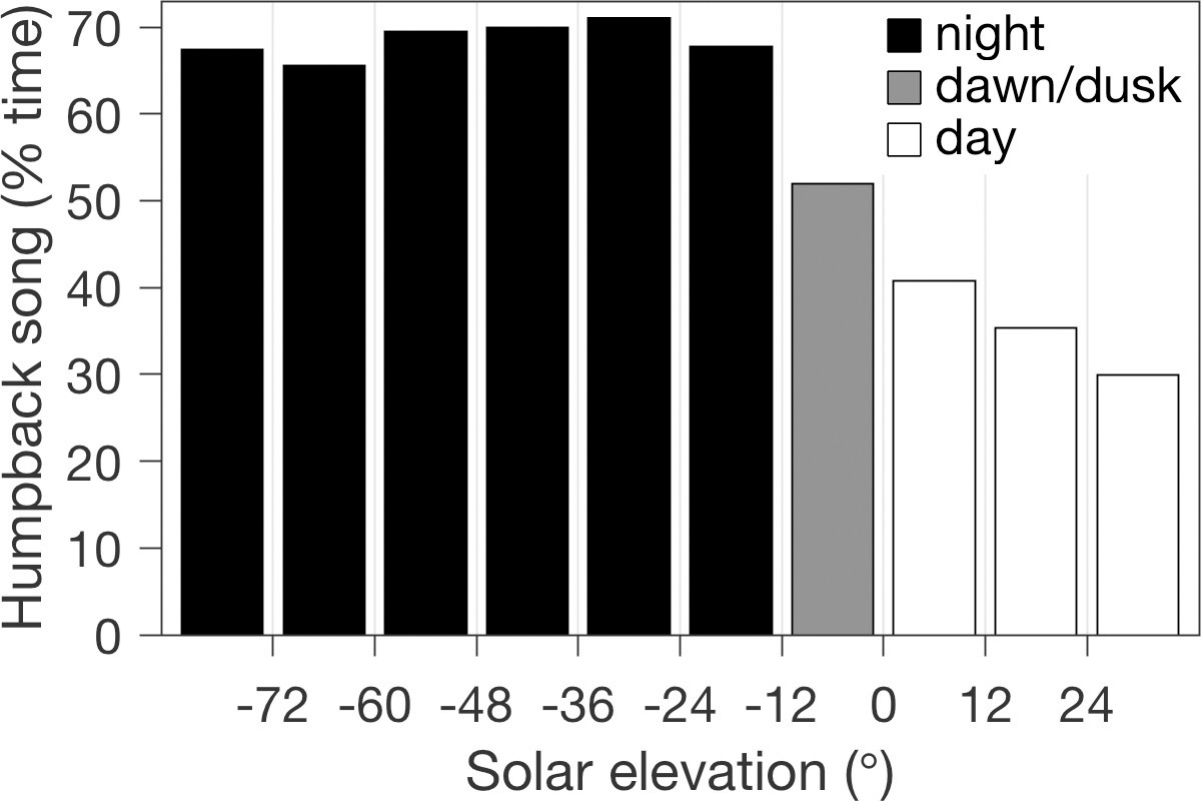
Diel variation in song occurrence. Values represent the percentage of recording time within each solar elevation bin during which song sessions were detected. This analysis pools all November through January data from the first three years of recordings (Fig 4), when 76% of all song was detected (Fig 5A).

As described in the methods, it is appropriate to constrain examination of diel variation to the time of year when song is prevalent. During the annual peak in song occurrence, November through January (Fig 5A), diel variation is pronounced (Fig 6). Maximum song occurrence is during the night, averaging 69% temporal coverage. Song occurrence decreases during dawn and dusk (52%), and further decreases with increasing solar elevation during the day to reach a minimum near solar noon (30%). Decreasing song occurrence with increasing solar elevation (Fig 6) is strongly linear (Model 1 linear regression, r^2^ = 0.99). Our results on diel variation are consistent with those from the study in the HCS [17]. In that study, when song detection was elevated (April—June), the highest levels were during dark and lowest levels were during daylight. Observations in breeding habitat around Hawaii have shown diel variation in song occurrence, maximum at night and minimum during the day, as well as increasing male competitive interactions during the day [74,75]. Alternative hypotheses for diel variation off Hawaii included whales singing louder or moving closer to the nearshore recorder during the night, or more whales singing at night [74]. Assuming more whales singing at night, the authors note that vision in daylight plays a key role in competitive group formation, and that males may switch to vocal advertisement as the primary mating strategy in darkness. These hypotheses proposed to explain diel variation in song occurrence within breeding habitat are relevant to its occurrence in the feeding and migratory habitat of our study. However, the importance of foraging activity to humpback whales in the Monterey Bay region introduces an additional consideration. If foraging strategies rely on vision in daylight, shifting behavior to foraging during daylight may reduce singing. It is not known whether humpbacks in this region sing in close spatiotemporal proximity to feeding behavior, as observed off Antarctica [16].

### Interannual

Interannual variation in humpback song occurrence was marked by 44% and 55% increases between consecutive years (Fig 4). Between the first and second years, the increase was associated with a longer temporal window of song detection between fall and spring, increasing from 197 to 233 days, and with greater occurrence during the peak months of November through January (Fig 4, Table 2). Between the second and third years, the increase was associated with yet greater occurrence during the peak months as well as a broader peak that started ~1 month earlier and ended ~1 month later (Fig 4, Table 2). Quantified on an annual basis, song temporal coverage increased from 11% in the first year to 25% in the third year, more than doubling during this period (Table 2). Alternative hypotheses for the interannual variation in song detection include interannual differences in the (a) local abundance of singing whales, (b) geographic distribution of singing whales in relation to the hydrophone location–in a way that would affect detection of song, (c) sound transmission to the hydrophone–as influenced by changes in hydrography, and (d) levels of singing activity, as may be influenced by animal health and time allocation to singing behavior. We consider these hypotheses using observational data and acoustic model results.

**Table 2:** Summary of temporal coverage and humpback song detection during the first two years of recordings.

	Year 1	Year 2	Year 3
Recording (days)	330.9 d	347.5 d	343.0 d
∑ song time (%)	11.2%	16.1%	25.1%
Detection window start and end, total days	10/12/2015 to 4/26/2016, 197 d	9/18/2016 to 5/9/2017, 233 d	9/5/2017 to 5/14/2018, 251 d
Song % of month, range	2.4% - 42.6%	0.3% - 56.8%	0.15% - 85.9%
Nov–Jan contribution to annual	83.2%	81.0%	70.9%

#### Environment

**Fig 7:**
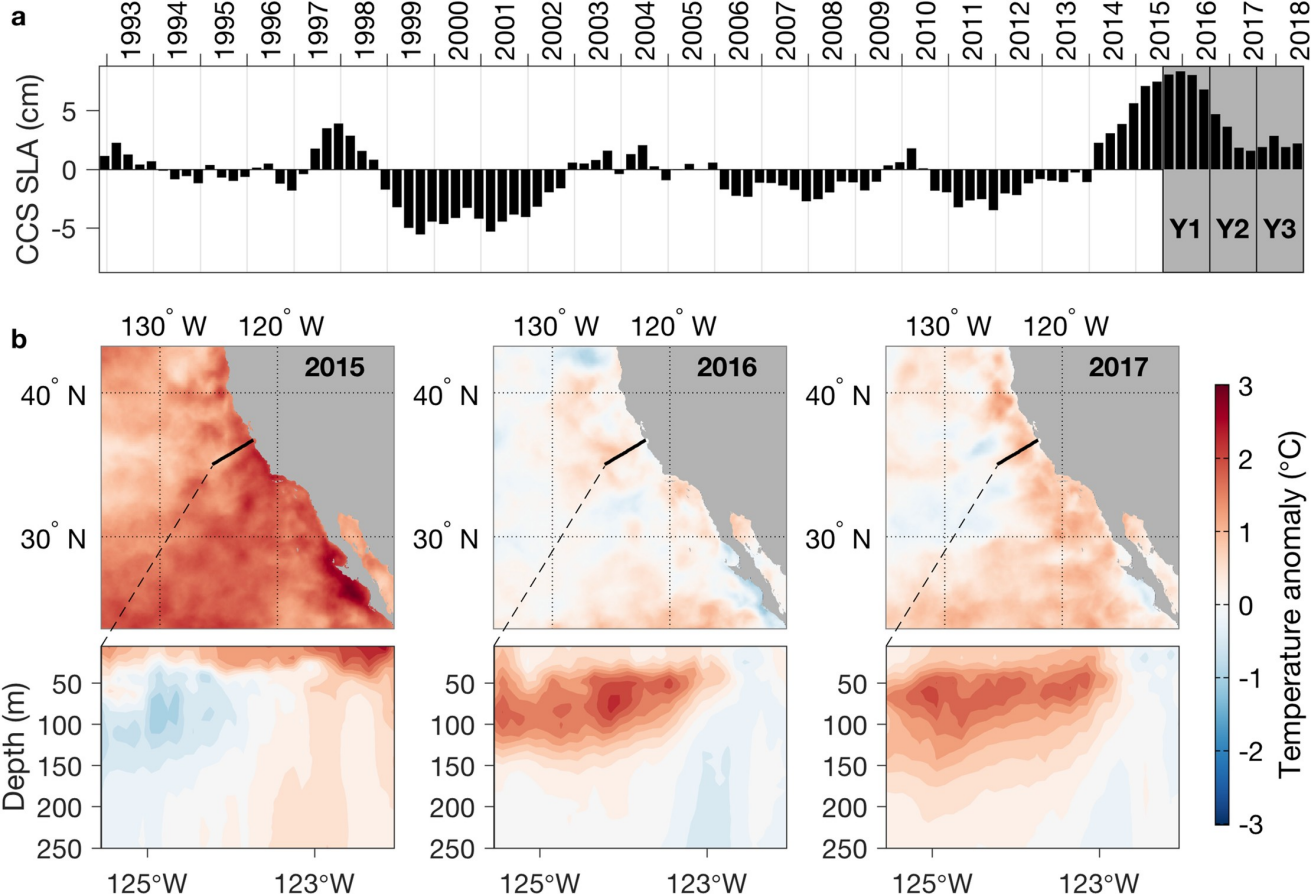
Interannual variations in the California Current System (CCS). (a) Sea level anomalies (SLA) averaged across the CCS. Sequential years of MARS recordings are indicated (Y1-Y3). (b) The peak and declining phases of a marine heat wave: surface (top) and subsurface (bottom) temperature anomalies during the second half of each calendar year. The black line in each map shows the mean path of the glider sampling that provided subsurface data for the vertical sections.

Consideration of all hypotheses for the strong interannual trend in song occurrence requires the context of interannual variations in ecosystem conditions. The first year of recording coincided with the highest sea level anomalies (SLA) in the CCS in the last 25 years (Y1 in Fig 7A). While SLA remained positive during the second and third years of recording, the magnitude of the anomaly was greatly reduced after the first year (Y2 and Y3 in Fig 7A). The exceptionally high SLA was due to a strong and persistent warm anomaly in the northeast Pacific [76]. Strongly positive SST anomalies prevailed throughout the CCS during 2015, and they ranged between weakly positive to weakly negative during 2016–2017 (Fig 7B). Subsurface temperature data extended between the coastal upwelling zone and the inner CCS off Monterey Bay (vertical sections in Fig 7B). These data show that the warm anomaly affected much of the upper water column across the oceanic margin, and that it increased toward the coast and toward the surface (Fig 7B, 2015). During 2016–2017 the near-coastal warm anomaly was absent (Fig 7B, east of ~123°W) although subsurface warm anomalies persisted further offshore. The major physical changes within ~ 80 km of the coast extended across primary foraging habitat of humpback whales.

#### Changes in local abundance of whales?

**Fig 8:**
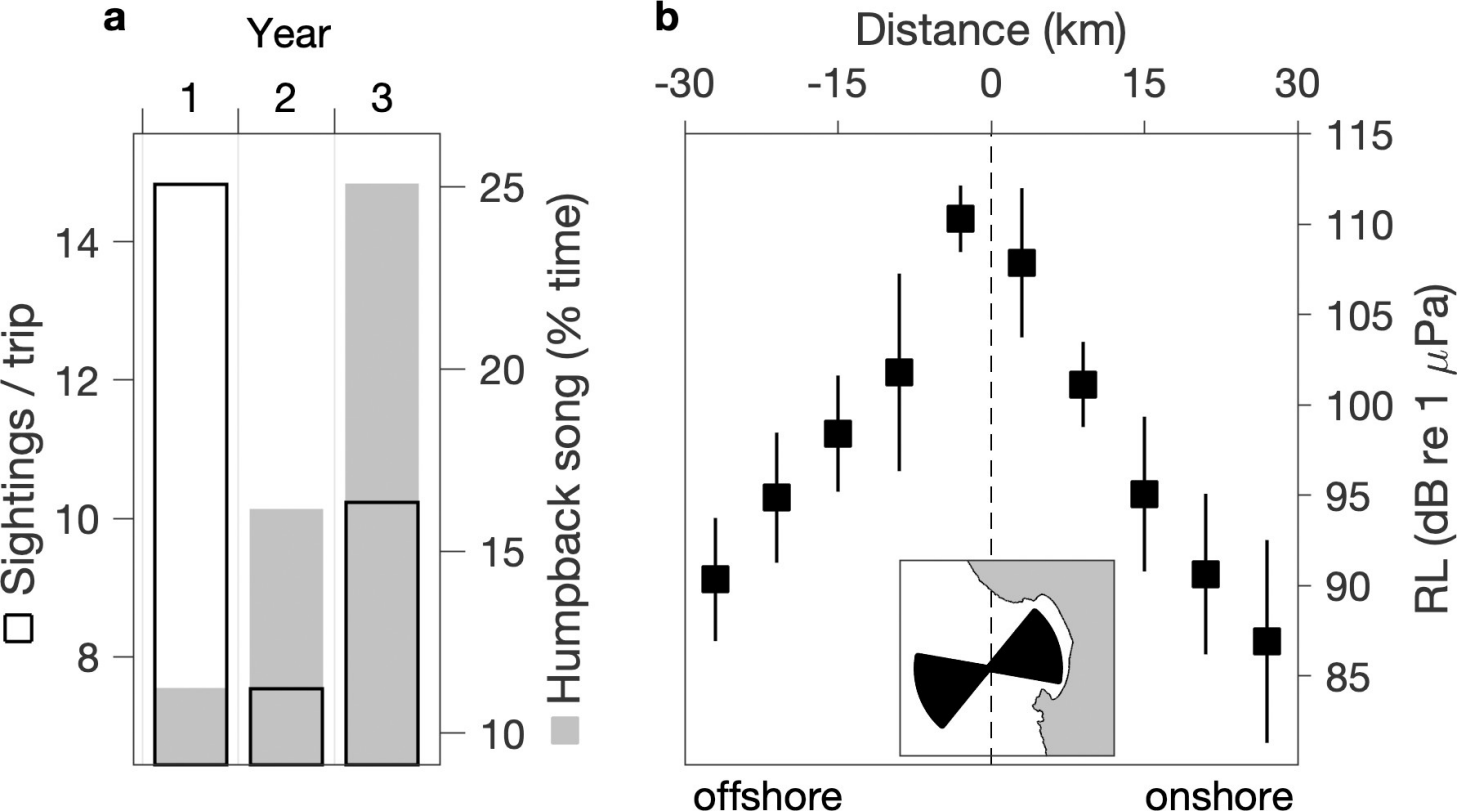
Potential causality of the interannual trend in humpback song occurrence from changes in local abundance and / or geographic distribution of whales. (a) Annual mean numbers of humpback whale sightings per whale-watching trip is compared with annual total song occurrence. (b) Mean and standard deviation of modeled received levels (RL; Fig 3A) within an onshore domain of 60° arc encompassing most of Monterey Bay and a mirrored offshore domain (inset map); distance bins are 6 km.

The first hypothesis for causality of the positive interannual trend in song is a positive trend in the local abundance of singing whales. Our sighting data from southern Monterey Bay are within the domain of MARS acoustic detection of humpback song (Fig 3A). Although our sighting data cannot constrain consideration to possible singing (adult male) whales, they represent extensive sampling. For the three years of recordings the sighting data span 82%, 82%, and 95%, respectively, of all days during months when song was detected—September through May (Fig 5A). Unlike the strong positive trend in song occurrence, sighting rates do not show a positive trend (Fig 8A). The increase in sightings between the second and third years may partly explain the increase in song detection between those years, however variation in the local abundance of whales is not indicated as causal in the song increase between the first and second years and cannot explain the steady positive trend over three years (Fig 8A). Further, coincidence of the lowest levels of song occurrence with the highest levels of sightings during the first year (Fig 8A) emphasizes the potential for behavioral regulation on the interannual time scale, as indicated at the seasonal time scale (Fig 5).

#### Changes in the geographic distribution of whales?

The second hypothesis is that interannual variation in song detection was influenced by changes in the geographic distribution of singing whales in relation to anomalous habitat conditions (Fig 7). A previous study suggests that rorqual whales, dominated by humpbacks, preferentially occupied habitat within Monterey Bay during a warm anomaly caused by El Niño and associated with reduced krill abundance [77]. Effects of the warm anomaly during our first year of recording share some similarity with El Niño effects, and a key question is whether preferential occupancy within Monterey Bay would reduce detection of song at the deep MARS site. Considering a 60° arc extending 30 km onshore from the hydrophone into Monterey Bay, mean received levels decrease 21 dB re 1 μPa between the hydrophone and inner shelf waters (Fig 8B). For a similar domain mirrored in the offshore direction, mean received levels decrease 20 dB re 1 μPa (Fig 8B). Thus, attenuation of song signal between singing whales and the hydrophone would not have been significantly greater for whales in Monterey Bay compared to offshore. These results do not support the hypothesis that preferential occupancy within Monterey Bay, if it occurred during the severe warm anomaly in first year of recording, would have reduced detection of song at the MARS site and thereby contributed to the positive interannual trend.

#### Changes in the acoustic transmission of whale song?

The third hypothesis is that changes in hydrography during this highly anomalous period (Fig 7) may have affected acoustic transmission to the hydrophone. If pycnocline depth was anomalously deep during the warm anomaly, relatively more song may have originated above the pycnocline and encountered greater attenuation due to the pycnocline. This is particularly relevant to song originating inside the bay, which would have a shallow angle of incidence relative to the hydrophone. Also, if pycnocline density gradients were anomalously strong during the warm anomaly, transmission loss may have increased, in turn reducing song detection at MARS. The potential role of variation in the pycnocline was evaluated using density profiles from stations M1 and C1 (white diamonds in Monterey Bay in Fig 1B). Although water density was anomalously low due to presence of the warm anomaly during the first year, the magnitude and vertical distribution of pycnocline density gradients was not anomalous. At both locations the maximum average vertical density gradient was shallower than 30 m during the song periods of all three years. Ship CTD profiles at the inner bay station were infrequent and inconsistent, with 8, 4, and 3 profiles during the song periods of each year, respectively. In contrast, CTD data from M1, at the mouth of Monterey Bay (middle white diamond in Fig 1B), was continuously recorded throughout all three song periods and is a better resource for comparison of magnitude. Across the three annual song periods, the magnitudes of the maximum average vertical density gradient were similar: 0.016, 0.016, and 0.018 kg m^-4^, respectively. The similarity in the depth and magnitude of pycnocline gradients across years indicates that hydrographic variation was unlikely to have influenced interannual variation in song detection at MARS.

#### Changes in singing behavior in relation to ecosystem variations?

**Fig 9:**
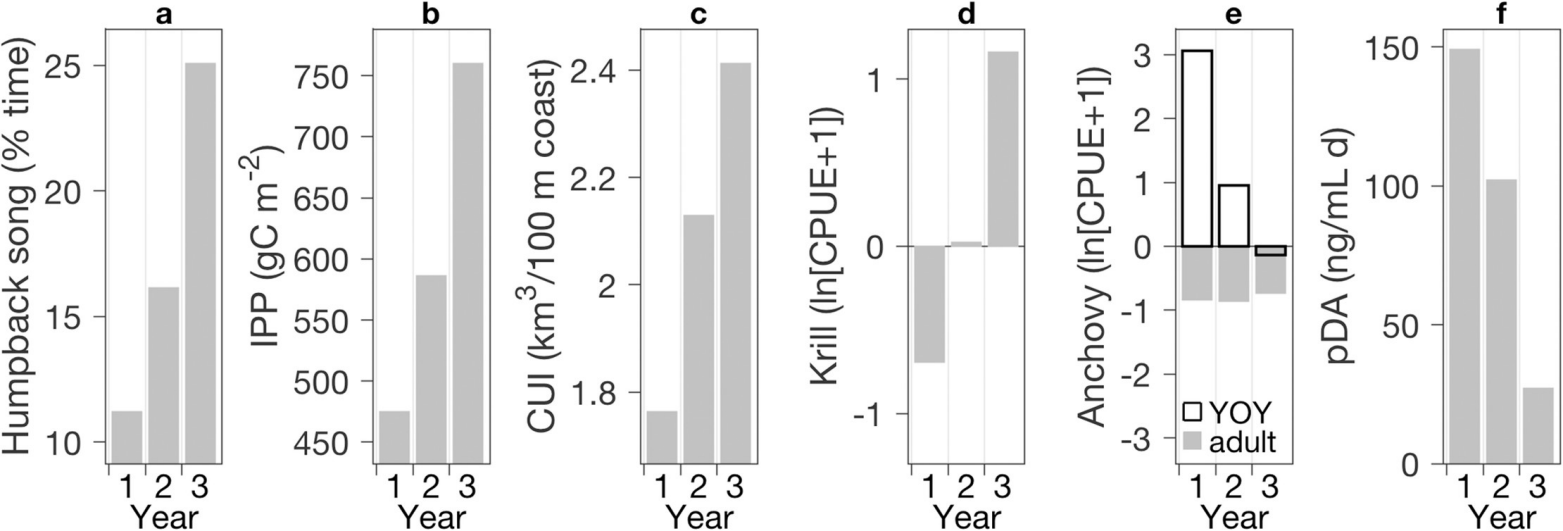
Annual metrics for (a) humpback song occurrence; (b) integrated primary productivity (station locations in Fig 1B); (c) cumulative upwelling; (d,e) krill and anchovy abundance anomalies from spring/summer surveys (average of data from red station locations in Fig 1B); (f) cumulative potential exposure to the neurotoxin domoic acid in the particulate fraction (pDA, available for trophic transfer) from weekly measurements at Santa Cruz Wharf (location in Fig 1B).

Extensive observations show major ecosystem variations relevant to humpback whale ecology. Further, these variations exhibit trends consistent with the observed trend in song occurrence. The trend of integrated primary productivity (IPP) in the Monterey Bay region closely parallels that of song occurrence (Fig 9A and 9B). IPP increased 60% between the first and third years (Fig 9B). Through trophic linkages in the short food chains between phytoplankton and baleen whales, such large changes in primary productivity could influence food resources for humpback whales. The trend in primary productivity followed that of cumulative upwelling (Fig 9C), i.e. wind-forced nutrient supply to the euphotic zone. It is also consistent with sea level variation (Fig 7A). Elevated sea level corresponds to a deeper thermocline (nutricline) and would cause relatively low levels of nutrient recruitment from wind-driven upwelling [78]. The decreasing trend in sea level thus represents shoaling of the nutricline and a positive trend in potential upwelling nutrient supply.

Consistent with the increasing trend in primary productivity, krill transitioned from a strong negative anomaly in the first year, to near normal in the second year, to a strong positive anomaly in the third year (Fig 9D). Adult anchovies exhibited depressed abundances during all three years (Fig 9E). The warm anomaly of the first year coincided with a large positive anomaly in young-of-year (YOY) anchovy (Fig 9E). One way these biological anomalies may have influenced humpback whale ecology is through prey switching. Large scale climatic variations in the CCS cause changes in the abundance and diversity of forage species [66,68], which in turn leads to prey switching in many species, including humpback whales [69]. If the forage species anomalies increased temporal and energetic demands of foraging, time and caloric energy available for singing behavior may have decreased.

Beyond the magnitude of primary productivity, a potentially influential factor is the extent to which primary productivity generates algal toxins that can affect marine mammal health and behavior. Our first year of recording coincided with a severe toxic algal bloom. Detection of the toxin, domoic acid (DA), in marine mammals—including whales, dolphins, porpoises, seals, and sea lions—spanned the largest geographic extent ever recorded [61]. Concentrations of DA in the particulate fraction (pDA), essential to trophic transfer of toxin, reached the highest levels ever recorded in Monterey Bay [79]. At a fixed location in northern Monterey Bay from which weekly monitoring is conducted (Fig 1B), integrated pDA was highest leading into and during this first year of recording, and it exhibited a decreasing trend thereafter (Fig 9F). Between the first and third years, integrated pDA decreased by 82%, and similar trends existed in the temporal span of pDA detection and maximum pDA concentrations (Fig 9F, Table 3). This neurotoxin can cause disease and mortality in marine mammals, including baleen whales [55,58–61]. Coincidence of highest potential DA exposure (Fig 9F) with lowest song activity amid the highest levels of humpback sightings (Fig 8A) suggests the possibility that high levels of DA exposure may suppress singing behavior.

**Table 3:** Summary of weekly particulate domoic acid (pDA) measurements at Santa Cruz Wharf (location in Fig 1B).

	2015	2016	2017
First and last detection date, span (days)	Apr 8 to Nov 25 231 days	Apr 20 to Oct 19 182 days	Mar 29 to Jun 28 91 days
Maximum concentration (ng/mL)	6.63	2.44	1.16
Integrated impact (ng/mL d)	148.9	102.1	27.4

## Conclusions

**Fig 10:**
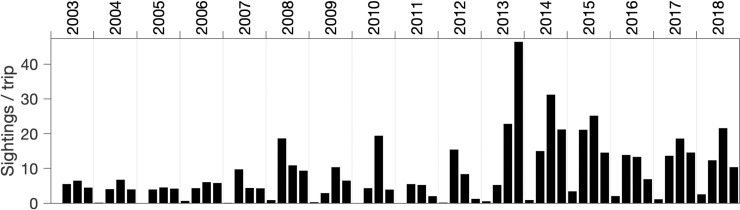
Quarterly mean sightings of humpback whales in Monterey Bay between 2003 and 2018, based on average sampling rates of 320 days per year.

This study extends the growing body of observations showing occurrence of humpback song in feeding and migratory habitats. In the northeast Pacific, the Monterey Bay region is important feeding and migratory habitat for humpback whales. The abundance of humpback whales off the U.S. West Coast has increased from about 900 individuals in the early–mid 1990s to about 2,900 whales in 2014, as this species has recovered from historic whaling impacts [80]. Sighting data indicate increased humpback whale abundances in Monterey Bay since 2013 (Fig 10), further supporting the critical role of this biologically important area [40] to humpback whale populations of the northeast Pacific.

Levels of song occurrence in this feeding and migratory habitat are high, occupying 9 months of the year with temporal coverage reaching as high as 86% of the time during the annual winter peak. Absence of song during summer, when visual sighting levels are high, emphasizes behavioral regulation of song activity. Coincidence of the annual trough in day length with both the annual song peak and evidence of population movement out of Monterey Bay is consistent with the underlying seasonal ecosystem variations and associated migratory patterns required for successful foraging and reproduction. These seasonal patterns observed in the northeastern Pacific are similar to those observed for humpback whales in the southeastern Pacific, northeastern Atlantic, and northwestern Atlantic. The rise of song activity leading into the breeding season is consistent with a central role in reproductive ecology, yet the prevalence of song in feeding and migratory habitats motivates further consideration of the greater spectrum of possible roles in social interaction, cultural transmission, and perception. Favorable foraging habitats that support relatively dense populations of whales may enhance density-dependent influences on song development and learning.

Interannual variation in song was great, exhibiting a positive trend over three years during which the temporal coverage of song doubled. Espousing the method of multiple working hypotheses [81], and within the limits of model and observational data, we considered alternative hypotheses. The hypothesis having some support is that exceptionally great physical and biological variations in the ecosystem influenced singing behavior, perhaps through changes in foraging ecology. While examination of causality between complex ecosystem variation and singing behavior can only be speculative, possible influences of the observed biological anomalies on humpback whale singing behavior are consistent with the observed changes in song detection. During the first year of recording, song levels were the lowest despite having the highest rate of humpback sightings in an area within the acoustic detection range of the hydrophone. This coincided with the greatest physical anomalies in the CCS in the last quarter century, severe depletion of a primary food resource–krill, and extremely high levels of a neurotoxin known to harm whale health–domoic acid. Relaxation of these extreme anomalies over the following two years, likewise, may have resulted in increased song activity by supporting greater health and reducing the time and energy requirements of foraging.

NOAA's National Marine Sanctuaries and National Marine Fisheries Service play a central role in understanding and protecting species and their habitats, with increasing interest in natural and anthropogenic sounds [82]. Passive acoustic monitoring is an essential and effective method of studying variations in marine mammal presence and behavior. The new knowledge of humpback whale song activity from this study has management implications, for example informing decisions on when to allow anthropogenic sounds through permitting processes. Interdisciplinary observations of complex marine ecosystems are essential to understanding ecosystem health and its long-term changes [83]. The integrated sound and ecosystem analysis presented in this study provides an example approach to comparative studies across regions having different mammal species, conservation threats, and opportunities to augment stewardship of protected species and habitats.

## References

[1] Payne RS, McVay S. Songs of Humpback Whales. Science. 1971; 173 (3997): 585–597. 10.1126/science.173.3997.585 17833100

[2] Cholewiak DM, Sousa‐Lima RS, Cerchio S. Humpback whale song hierarchical structure: Historical context and discussion of current classification issues. Mar Mamm Sci. 2013; 29:E312–E332.

[3] Frankel AS, Clark CW, Herman LM, Gabriele CM. Spatial distribution, habitat utilization, and social interactions of humpback whales, *Megaptera novaeangliae*, off Hawai’i, determined using acoustic and visual techniques. Can J Zool. 1995; 73, 1134–1146.

[4] Smith JN, Goldizen AW, Dunlop RA, Noad MJ. Songs of male humpback whales, *Megaptera novaeangliae*, are involved in intersexual interactions. Animal Behav. 2008; 76(2): 467–477. 10.1016/j.anbehav.2008.02.013

[5] Cholewiak DM, Cerchio S, Jacobsen JK, Urbán-R. J, Clark CW. Songbird dynamics under the sea: acoustic interactions between humpback whales suggest song mediates male interactions. R Soc Open Sci. 2018; 10.1098/rsos.171298 29515847 PMC5830736

[6] Norris TF, McDonald M, Barlow J. Acoustic detections of singing humpback whales (*Megaptera novaeangliae*) in the eastern North Pacific during their northbound migration. J Acoust Soc Am. 1999; 106:506–514. 10.1121/1.427071 10420640

[7] Mercado E III. The Sonar Model for Humpback Whale Song Revised. Front Psychol. 2018; 9:1156 10.3389/fpsyg.2018.01156 30061851 PMC6055024

[8] Noad MJ, Cato DH, Bryden MM, Jenner M, Jenner KCS. Cultural revolution in whale songs. Nature. 2000; 408:537 10.1038/35046199 11117730

[9] Garland EC, Goldizen AW, Rekdahl ML, Constantine R, Garrigue C, Hauser ND, et al Dynamic horizontal cultural transmission of humpback whale song at the ocean basin scale. Curr Biol. 2011; 21(8):687–691. 10.1016/j.cub.2011.03.019 21497089

[10] Fournet MEH, Gabriele CM, Culp DC, Mellinger DK, Sharpe F, Klinck H. Some things never change: multi-decadal stability in humpback whale calling repertoire on Southeast Alaskan foraging grounds. Sci Rep. 2018; 8(1): 13186 10.1038/s41598-018-31527-x 30262835 PMC6160409

[11] Mattila DK, Guinee LN, Mayo CA. Humpback whale songs on a North Atlantic feeding ground. J Mammal. 1987; 68(4): 880–883.

[12] McSweeney DJ, Chu KC, Dolphin WF, Guinee, LN. North Pacific humpback whale songs: a comparison of southeast Alaskan feeding ground songs with Hawaiian wintering ground songs. Mar Mamm Sci. 1989; 5(2):139–148. 10.1111/j.1748-7692.1989.tb00328.x

[13] Charif RA, Clapham PJ, Clark CW. Acoustic detections of singing humpback whales in deep waters off the British Isles. Mar Mamm Sci. 2001; 17(4): 751–768. 10.1111/j.1748-7692.2001.tb01297.x

[14] Clark CW, Clapham PJ. Acoustic monitoring on a humpback whale (*Megaptera novaeangliae*) feeding ground shows continual singing into late spring. Proc R Soc Lond B. 2004; 271:1051–1057. 10.1098/rspb.2004.2699 15293859 PMC1691688

[15] Vu ET, Risch D, Clark CW, Gaylord S, Hatch LT, Thompson MA, et al Humpback whale song occurs extensively on feeding grounds in the western North Atlantic Ocean. Aquat Biol. 2012; 14:175–183. 10.3354/ab00390

[16] Stimpert AK, Peavey LE, Friedlaender AS, Nowacek DP. Humpback whale song and foraging behavior on an Antarctic feeding ground. PLoS ONE. 2012; 7(12): e51214 10.1371/journal.pone.0051214 23284666 PMC3526533

[17] Español-Jiménez S, van der Schaar M. First record of humpback whale songs in Southern Chile: Analysis of seasonal and diel variation. Mar Mamm Sci. 2018; 34(3):718–733. 10.1111/mms.12477

[18] Kowarski K, Evers C, Moors‐Murphy H, Martin B, Denes SL. Singing through winter nights: Seasonal and diel occurrence of humpback whale (*Megaptera novaeangliae*) calls in and around the Gully MPA, offshore eastern Canada. Mar Mamm Sci. 2018; 34: 169–189. 10.1111/mms.12447

[19] Magnúsdóttir EE, Lim R. Subarctic singers: Humpback whale (*Megaptera novaeangliae*) song structure and progression from an Icelandic feeding ground during winter. PLoS ONE. 2019; 14(1): e0210057 10.1371/journal.pone.0210057 30673737 PMC6343865

[20] Clapham PJ, Mattila DK. Humpback whale songs as indicators of migration routes. Mar Mamm Sci. 1990; 6: 155–160. 10.1111/j.1748-7692.1990.tb00238.x

[21] Dawbin WH, Gill PC. Humpback whale survey along the west coast of Australia: a comparison of visual and acoustic observations. Mem Qld Mus. 1991; 30: 255–257.

[22] Bettridge S, Baker CS, Barlow J, Clapham PJ, Ford M, Gouveia D, et al Status review of the humpback whale (*Megaptera novaeangliae*) under the endangered species act. NOAA -TM-NMFS-SWFSC-540. 2015. U. S. Department of Commerce.

[23] Baker CS, Palumbi SR, Lambertsen RH, Weinrich MT, Calambokidis J, O’Brien SJ. Influence of seasonal migration on the distribution of mitochondrial DNA haplotypes in humpback whales. Nature. 1990; 344:238–240. 10.1038/344238a0 1969116

[24] Baker CS, Slade RW, Bannister JL, Abernethy RB, Weinrich MT, Lien J, et al Hierarchical structure of mitochondrial DNA gene flow among humpback whales *Megaptera novaeangliae*, world-wide. Mol Ecol. 1994; 3:313–327. 7921358 10.1111/j.1365-294x.1994.tb00071.x

[25] Baker CS, Steel D, Calambokidis J, Falcone E, González-Peral U, Barlow J, et al Strong maternal fidelity and natal philopatry shape genetic structure in North Pacific humpback whales. Mar Ecol Prog Ser. 2013; 494:291–306. 10.3354/meps10508

[26] Steiger GH, Calambodikis J, Sears R, Balcomb KC, Cubbage JC. Movement of humpback whales between California and Costa Rica. Mar Mamm Sci. 1991; 7:306–310. 10.1111/j.1748-7692.1991.tb00105.x

[27] Calambokidis J, Steiger GH, Evenson JR, Flynn KR, Balcomb KC, Claridge DE, et al Interchange and isolation of humpback whales off California and other feeding grounds in the North Pacific. Mar Mamm Sci. 1996; 12:215–226. 10.1111/j.1748-7692.1996.tb00572.x

[28] Calambokidis J, Steiger GH, Rasmussen K, Urbán J, Balcomb KC, Ladrón de Guevara P, et al Migratory destinations of humpback whales that feed off California, Oregon and Washington. Mar Ecol Prog Ser. 2000; 192:295–304. 10.3354/meps192295

[29] Urbán J, Jaramillo A, Aguayo A, Ladrón de Guevara P, Salinas M, Alvarez C, et al Migratory destinations of humpback whales wintering in the Mexican Pacific. J Cetacean Res Manag. 2000; 2: 101–110.

[30] Calambokidis J, Barlow J. Abundance of blue and humpback whales in the eastern North Pacific estimated by capture‐recapture and line‐transect methods. Mar Mamm Sci. 2004; 20: 63–85. 10.1111/j.1748-7692.2004.tb01141.x

[31] Online: https://sanctuarysimon.org/1970/02/historic-whaling-in-moss-landing/.

[32] Ryther JH. Photosynthesis and Fish Production in the Sea. Science. 1969; 166 (3901): 72–76. 10.1126/science.166.3901.72 5817762

[33] Huyer A. Coastal upwelling in the California Current System. Prog Oceanogr. 1983; 12:259–284. 10.1016/0079-6611(83)90010-1

[34] Barber RT, Smith RL. Coastal upwelling ecosystems. In: Longhurst AR, editor. Analysis of Marine Ecosystems. Academic; 1981 pp. 31–68.

[35] Carr ME, Kearns EJ. Production regimes in four Eastern Boundary Current systems. Deep Sea Res Part II Top Stud Oceanogr. 2003; 50:3199–3221. 10.1016/j.dsr2.2003.07.015

[36] Chavez FP, Messié M. A comparison of Eastern Boundary Upwelling Ecosystems. Prog Oceanogr. 2009; 83: 80–96. 10.1016/j.pocean.2009.07.032

[37] Santora JA, Sydeman WJ, Schroeder ID, Wells BK, Field JC. Mesoscale structure and oceanographic determinants of krill hotspots in the California Current: Implications for trophic transfer and conservation. Prog Oceanogr. 2011; 91:397–409. 10.1016/j.pocean.2011.04.002

[38] Santora JA, Field JC, Schroeder, Sakuma KM, Wells BK, Sydeman WJ. Spatial ecology of krill, micronekton and top predators in the central California Current: Implications for defining ecologically important areas. Prog Oceanogr. 2012; 106:154–174. 10.1016/j.pocean.2012.08.005

[39] Santora JA, Zeno R, Dorman JG, Sydeman WJ. Submarine canyons represent an essential habitat network for krill hotspots in a Large Marine Ecosystem. Sci Rep. 2018; 8(1). 10.1038/s41598-018-25742-9 29765085 PMC5954138

[40] Calambokidis J, Steiger GH, Curtice C, Harrison J, Ferguson MC, Becker EA, et al Biologically important areas for selected cetaceans within U.S. waters ‐ West Coast region. Aquat Mamm. 2015; 41(1): 39–53. 10.1578/AM.41.1.2015.39

[41] Becker EA, Forney KA, Fiedler PC, Barlow, Chivers SJ, Edwards CA, et al Moving Towards Dynamic Ocean Management: How Well Do Modeled Ocean Products Predict Species Distributions? Remote Sens. 2016; 8:149 10.3390/rs8020149

[42] Becker EA, Forney KA, Redfern JV, Barlow J, Jacox MG, Roberts JJ, et al Predicting cetacean abundance and distribution in a changing climate. Divers Distrib. 2019; 25: 626–643. 10.1111/ddi.12867

[43] Ryan J, Cline D, Dawe C, McGill P, Zhang Y, Joseph J, et al New passive acoustic monitoring in Monterey Bay National Marine Sanctuary OCEANS MTS/IEEE, Monterey, CA 2016; pp. 1–8. 10.1109/OCEANS.2016.7761363

[44] Online: cetus.ucsd.edu/technologies_Software.html.

[45] Online: http://www.cs.wisc.edu/condor.

[46] Online: naif.jpl.nasa.gov/naif/.

[47] Collins MD. A split-step Padé solution for the parabolic equation method. J Acoust Soc Am. 1993; 93:1736–1742. 10.1121/1.406739 35232107

[48] Au WWL, Pack AA, Lammers MO, Herman LM, Deakos MH, Andrews K. Acoustic properties of humpback whale songs. J Acoust Soc Am. 2006; 120(2):1103–1110. 10.1121/1.2211547 16938996

[49] Online: https://podaac.jpl.nasa.gov/Integrated_Multi-Mission_Ocean_AltimeterData.

[50] Auad G, Roemmich D, Gilson J. The California Current System in relation to the Northeast Pacific Ocean circulation. Prog Oceanogr. 2011; 91:576–592.

[51] Schwing FB, O’Farrell M, Steger JM, Baltz K. Coastal upwelling indices west coast of North America 1946–95. NOAA-TM-NMFS-SWFSC-231 1996 U.S. Department of Commerce, National Oceanic and Atmospheric Administration, National Marine Fisheries Service.

[52] Online: https://coastwatch.pfeg.noaa.gov/erddap.

[53] Rudnick DL, Zaba KD, Todd RE, Davis RE. A climatology of the California Current System from a network of underwater gliders. Prog Oceanogr. 2017; 154:64–106. 10.1016/j.pocean.2017.03.002

[54] Pennington JT, Chavez FP. Seasonal fluctuations of temperature, salinity, nitrate, chlorophyll and primary production at station H3/M1 over 1989–1996 in Monterey Bay, California. Deep Sea Res Part II Top Stud Oceanogr. 2000; 47(5–6):947–973. 10.1016/S0967-0645(99)00132-0

[55] Scholin CA, Gulland F, Doucette GJ, Benson S, Busman M, Chavez FP, et al Mortality of sea lions along the central California coast linked to a toxic diatom bloom. Nature. 2000; 403, 80–84. 10.1038/47481 10638756

[56] Trainer VL, Bates SS, Lundholm N, Thessen AE, Adams NG, Cochlan WP, et al *Pseudo‐nitzschia* physiological ecology, phylogeny, toxicity, monitoring and impacts on ecosystem health. Harmful Algae. 2012; 14, 271–300.

[57] Kudela R, Pitcher G, Probyn T, Figueiras F, Moita T, Trainer V. Harmful algal blooms in coastal upwelling systems. Oceanography. 2005; 18, 184–197.

[58] Van Dolah FM (2005) Effects of harmful algal blooms In: Reynolds J, Perrin W, Reeves R, Montgomery S, Ragen T, editors. Marine mammal research: conservation beyond crisis. Johns Hopkins University Press; 2005 pp. 85–101.

[59] Fire SE, Wang Z, Leighfield TA, Morton SL, McFee WE, McLellan WA, et al Domoic acid exposure in pygmy and dwarf sperm whales (*Kogia* spp.) from southeastern and mid-Atlantic U.S. waters. Harmful Algae. 2009; 8(5):658–664. 10.1016/j.hal.2008.12.002

[60] Fire SE, Wang Z, Berman M, Langlois GW, Morton SL, Sekula-Wood E, et al Trophic transfer of the harmful algal toxin domoic acid as a cause of death in a minke whale (*Balaenoptera acutorostrata*) stranding in southern California. Aquat Mamm. 2010; 36(4):342–350. 10.1578/AM.36.4.2010.342

[61] McCabe RM, Hickey BM, Kudela RM, Lefebvre KA, Adams NG, Bill BD, et al An unprecedented coastwide toxic algal bloom linked to anomalous ocean conditions. Geophys Res Lett. 2016; 43, 10,366–10,376, 10.1002/2016GL070023 27917011 PMC5129552

[62] Lane JQ, Roddam CM, Langlois GW, Kudela RM. Application of Solid Phase Adsorption Toxin Tracking (SPATT) for field detection of the hydrophilic phycotoxins domoic acid and saxitoxin in coastal California. Limnol Oceanogr Methods. 2010; 8, 645–660.

[63] Online: http://sccoos.org.

[64] Sakuma KM, Field JC, Mantua NJ, Ralston S, Marinovic BB, Carrion CN. Anomalous epipelagic micronekton assemblage patterns in the neritic waters of the California Current in spring 2015 during a period of extreme ocean conditions. CalCOFI Rep. 2016; 57: 163−183.

[65] Ralston S, Field JC, Sakuma KM. Long-term variation in a central California pelagic forage assemblage. J Mar Syst. 2015; 146: 26−37.

[66] Santora JA, Hazen EL, Schroeder ID, Bograd SJ, Sakuma KM, Field JC. Impacts of ocean climate variability on biodiversity of pelagic forage species in an upwelling ecosystem. Mar Ecol Prog Ser. 2017; 580:205–220. 10.3354/meps12278

[67] Szoboszlai AI, Thayer JA, Wood SA, Sydeman WJ, Koehn LE. Forage species in predator diets: synthesis of data from the California Current. Ecol Inform. 2015; 29:45–56.

[68] Santora JA, Schroeder ID, Field JC, Wells BK, Sydeman WJ. Spatio-temporal dynamics of ocean conditions and forage taxa reveals regional structuring of seabird−prey relationships. Ecol Appl. 2014; 24: 1730−1747. 29210234 10.1890/13-1605.1

[69] Fleming AH, Clark CT, Calambokidis J, Barlow J. Humpback whale diets respond to variance in ocean climate and ecosystem conditions in the California Current. Glob Change Biol. 2016; 22: 1214–1224. 10.1111/gcb.13171 26599719

[70] Online: https://www.integratedecosystemassessment.noaa.gov/regions/california-current/cc-indicator-status-trends.

[71] Thompson AR, Schroeder ID, Bograd SJ, Hazen EL, Jacox MG, Leising A, et al (2018) State of the California Current, 2017–18 CalCOFI Rep. 2018; 59 California Cooperative Oceanic Fisheries Investigations. University of California, San Diego.

[72] Wright A, Walsh L. Mind the gap: Why neurological plasticity may explain seasonal interruption in humpback whale song. J Mar Biol Assoc U.K. 2010; 90(8):1489–1491. 10.1017/S0025315410000913

[73] Cates KA, Atkinson S, Gabriele CM, Pack AA, Straley JM, Yin S. Testosterone trends within and across seasons in male humpback whales (*Megaptera novaeangliae*) from Hawaii and Alaska. Gen Comp Endocrinol. 2019; 279:164–173. 10.1016/j.ygcen.2019.03.013 30904390

[74] Au WW, Mobley J, Burgess WC, Lammers MO, Nachtigall PE. Seasonal and diurnal trends of chorusing humpback whales wintering in waters off western Maui. Mar Mamm Sci. 2000; 16: 530–544. 10.1111/j.1748-7692.2000.tb00949.x

[75] Helweg DA, Herman LM. Diurnal patterns of behaviour and group membership of humpback whales (*Megaptera novaeangliae*) wintering in Hawaiian waters. Ethology. 1994; 98: 298–311. 10.1111/j.1439-0310.1994.tb01078.x

[76] Gentemann CL, Fewings MR, García-Reyes M. Satellite sea surface temperatures along the west coast of the United States during the 2014–2016 northeast Pacific marine heat wave. Geophys Res Lett. 2017; 44:312–319. 10.1002/2016GL071039

[77] Benson SR, Croll DA, Marinovic BB, Chavez FP, Harvey JT. Changes in the cetacean assemblage of a coastal upwelling ecosystem during El Niño 1997–98 and La Niña 1999. Prog Oceanogr. 2002; 54:279–291. 10.1016/S0079-6611(02)00054-X.

[78] Chavez FP, Pennington JT, Castro CG, Ryan JP, Michisaki RP, Schlining B, et al Biological and chemical consequences of the 1997–98 El Niño in central California waters. Prog Oceanogr. 2002; 54:205–232.

[79] Ryan JP, Kudela RM, Birch JM, Blum M, Bowers HA, Chavez FP, et al Causality of an extreme harmful algal bloom in Monterey Bay, California, during the 2014–2016 northeast Pacific warm anomaly. Geophys Res Lett. 2017; 44: 5571–5579. 10.1002/2017GL072637

[80] Calambokidis J, Barlow J, Flynn K, Dobson E, Steiger GH. Update on abundance, trends, and migrations of humpback whales along the US West Coast. 2017 International Whaling Commission Paper SC/A17/NP/13. 17 pp.

[81] Chamberlin TC. The method of multiple working hypotheses. Science. 1890; reprinted 1965; 148(3671): 754–759. 10.1126/science.148.3671.754 17748786

[82] Gedamke J, Harrison J, Hatch L, Angliss R, Barlow J, Berchok C, et al NOAA Ocean Noise Strategy Roadmap NOAA 2016; 138pp. Online: https://cetsound.noaa.gov/road-map.

[83] Office of National Marine Sanctuaries. Monterey Bay National Marine Sanctuary Condition Report partial update: a new assessment of the state of Sanctuary resources. 2015. U.S. Department of Commerce, NOAA, Office of National Marine Sanctuaries, Silver Spring, MD. 133 pp.

